# Hematological neoplasms after PRRT: experience from long term follow-up of 173 patients in a tertiary center

**DOI:** 10.1530/EO-26-0038

**Published:** 2026-08-17

**Authors:** Joao Pedro Thimotheo Batista, Michael De La Iglesia, Hyun Kim, Vikas Prasad, Ravindra Shubha, Rama Suresh, Shuang Wu, Jingxia Liu, Meagan Jacoby, Samuel Urrutia, Nikolaos A Trikalinos

**Affiliations:** ^1^ MetroWest Medical Center and Framingham Union Hospital, Framingham, USA; ^2^ Department of Medical Oncology, Washington University School of Medicine, St. Louis, Missouri, USA; ^3^ Department of Radiation Oncology, Washington University School of Medicine, St. Louis, Missouri, USA; ^4^ Department of Nuclear Medicine, Washington University School of Medicine, St. Louis, Missouri, USA; ^5^ Department of Surgery, Washington University School of Medicine, St. Louis, Missouri, USA

**Keywords:** PRRT, AML, MDS, neuroendocrine neoplasms, lutetium DOTATATE

## Abstract

**Objective:**

Peptide receptor radionuclide therapy (PRRT) with lutetium-177 DOTATATE (^177^Lu-DOTATATE) is widely adopted as a later-line treatment (second-line or subsequent therapy) for patients with somatostatin receptor-positive neuroendocrine neoplasm. The registration NETTER-1 trial reported a late-onset adverse event of therapy-related myeloid neoplasms (tMNs), including myelodysplastic syndrome (MDS)/acute myeloid leukemia (AML) in approximately 1% of patients. In this retrospective study, we sought to investigate the frequency of tMN in patients treated with ^177^Lu-DOTATATE at our institution over the last 8 years and determine the treatment characteristics of patients developing tMN.

**Methods:**

We accessed the treatment files of patients with neuroendocrine neoplasms treated at our institution between January 2017 and January 2025 for pathology reports of myelodysplasia, leukemia, aplastic anemia and similar hematological disorders.

**Results:**

A total of 173 patients received ^177^Lu-DOTATATE. A vast majority (77.5%) of them were treated with four therapy cycles. A total of 10/173 (5.8%) patients (4 males, 6 females) were diagnosed with tMN at a median of 23 months (5.63) after first cycle. One patient developed severe thrombocytopenia, stopped all treatments and was lost to follow-up. Two patients developed acute myelogenous leukemia (AML), six developed myelodysplastic syndrome (MDS), one developed aplastic anemia, and one developed multilineage dyspoiesis (the latter two also deemed to have MDS). Eight out of ten (80%) died after a median duration of 8 months (4.23) from tMN diagnosis. Two patients were diagnosed with AML after 2 and 4 years from the start of first cycle, while 40% of MDS cases were diagnosed by 1 year after cycle 1 of ^177^Lu-DOTATATE. Additionally, multilineage dyspoiesis was observed at around 5 years.

**Conclusions:**

The prevalence of tMN was 5.8% in our patient population. We have noted a higher than previously reported percentage of myelodysplasia post-PRRT, especially with additional treatments and longer follow-ups.

## Introduction

We cannot understate the cumulative impact of peptide receptor radionuclide therapy (PRRT) on the treatment options for patients with neuroendocrine neoplasms (NENs), especially given the reported progression-free survival (PFS) differences. ^177^Lu-DOTATATE is a radiolabeled somatostatin analog that delivers targeted beta-particle radiation to neuroendocrine tumor cells expressing somatostatin receptors. As this treatment is very well tolerated with arguably the best objective response rate, an increasing number of patients have opted for PRRT after progressing on somatostatin analogs since the approval of ^177^Lu-DOTATATE for grade 1 (G1) and G2 gastroenteropancreatic neuroendocrine tumors based on the landmark NETTER-1 study (1). The long-term safety profile of NETTER-1 raised no additional concerns (1). Subsequently, the NETTER-2 clinical trial showed efficacy of ^177^Lu-DOTATATE even in higher grade 3 NET with Ki-67 up to 55% (2). In another phase 3 clinical trial, the radiopharmaceutical Lu-177 DOTATOC targeting somatostatin receptor subtypes 2 and 5 is being investigated for higher-grade NETs (3). Since then, the field is getting more attention with trials exploring combination therapies (4), personalized dosimetry and promising alpha emitter-labeled somatostatin receptor-targeting peptides (5).

However, alongside the research for better, more effective radiation dosing, there is an urgent need to establish the long-term safety of targeted radiopharmaceutical therapies in real-world setting. Myelotoxicity, in the form of cytopenia, established myelodysplastic syndrome (MDS) or acute myelogenous leukemia (AML), happens at a variable, uncertain rate and with an equally uncertain lag period after treatment with PRRT. Patients who develop therapy-related myeloid neoplasms (tMNs) can live as little as 6 months (6), significantly shortening their lifespan. Moreover, even cytopenias can affect quality of life and impact patient’s eligibility for future treatments or clinical trials.

The knowledge of secondary tMNs after PRRT is limited. PRRT has a long history of use in Europe and Australia, but the available retrospective studies, allowing for variable long-term follow-up, have produced equally variable percentages. For example, the phase 3 trial NETTER-1 trial reported a tMN incidence of 2% after a median follow-up of 76.3 months (1); similar findings were noted in NETTER-2. A systematic review published in 2020, which analyzed 28 studies, found a mean tMN incidence of 2.61%, with follow-up durations ranging from 14 to 88 months (7). In contrast, studies with longer follow-up periods have observed higher incidence rates of tMNs (8, 9). It is plausible that shorter follow-up periods may contribute to underdiagnosis. However, while the follow-up duration varied across studies, no statistically significant association has been observed between a longer follow-up and an increased incidence of tMNs. Although prior treatment with alkylating chemotherapies, everolimus and combination of ^177^Lu-DOTATATE with chemotherapy have been reported to be associated with a higher incidence of tMNs, the exact causal relationship has not been established.

Recognizing these limitations, we hypothesized that the real-world incidence of tMNs following PRRT may be higher than currently recognized. Institutional databases from tertiary comprehensive cancer centers have long been considered a rich source for collecting long-term data to establish and explore the true prevalence of tMNs after ^177^Lu-DOTATATE. Our tertiary center sees >100 new patients/year and has been effectively using ^177^Lu-DOTATATE since its approval, with a smaller number of patients being treated before 2018 as part of an experimental protocol. It is also a major referral center for patients diagnosed with bone marrow disorders. In this retrospective chart-based study, we describe the frequency of tMNs after PRRT and outcome of such incidences (overall survival) and compare this experience to the published literature.

## Materials and methods

We performed a retrospective analysis of the long-term hematological adverse effects of patients with neuroendocrine neoplasms treated with ^177^Lu-DOTATATE. We obtained an approval from the Institutional Review Board (IRB) of Washington University in St Louis to access files of patients who had completed treatment between 01/2017 and 12/2022. We further identified incidences of myelodysplasia (MDS) or leukemia (AML or CML) from patient’s charts. All leukemia/MDS diagnoses were based on either a bone marrow biopsy or an expert consensus by the malignant hematology department. No contact was made with patients or relatives. For survival purposes/censoring, the last patient check was performed on 31 January, 2025, so patients were followed up for a theoretical minimum of 2 and maximum of 10 years. For the cohort of screened patients, we extracted age at PRRT initiation, sex, PRRT fractions, time to AML/MDS and death or last contact. For patients who were diagnosed with AML/MDS, we extracted the following: age, sex, race, date of initial diagnosis, NEN histologic characteristics, tumor volume by imaging, treatment details before and after PRRT, dosing details of PRRT, date of hematologic disorder diagnosis, genetic results (IHC and NGS) on NEN and marrow biopsies, and survival metrics.

Tumor volume was estimated from baseline pre-PRRT SSTR PET/CT using MIM software (MIM Software Inc., USA). Tumor segmentation and volumetric categorization were performed in accordance with the methodology described by Carlson *et al.* (10), including the use of a liver-based thresholding approach and predefined tumor volume cutoffs. For each patient, a 3 cm spherical volume of interest (VOI) was placed within normal liver parenchyma to obtain reference uptake values. The liver SUVmean and standard deviation (SD) were extracted and used to calculate a patient-specific segmentation threshold using the following formula: Threshold = 1.5 × liver SUVmean + 2 × liver SD. The standardized uptake value (SUV) was defined as the decay-corrected radioactivity concentration divided by injected activity normalized to body weight. A region encompassing all suspected lesions was manually delineated, and voxels with SUV values above the calculated threshold were automatically segmented to identify tumor tissue. Physiologic and non-pathologic tracer uptake – including the pituitary gland, liver, spleen, kidneys, adrenal glands, urinary tract and uncinate process of the pancreas – was carefully excluded. The remaining segmented voxels were used to calculate total tumor volume (TTV). Total lesion somatostatin receptor expression (TL-SSTR) was calculated as follows: TL-SSTR = SUVmean × TTV. Tumor volume was categorized as low (<54.9 mL) and high (≥54.9 mL).

### Statistical analysis

R version 4.4.2 (R Foundation for Statistical Computing, Vienna, Austria) was used to perform all statistical analyses. Demographic and clinical characteristics were summarized using descriptive statistics, including the median and range of continuous variables and frequencies and percentages of categorical variables. Competing risk analysis was conducted to account for MDS/AML as a competing event for death. Cumulative incidence functions were estimated at posttreatment time points for both death and MDS/AML. A multivariable competing risks regression model was constructed to estimate hazard ratios and 95% confidence intervals (CIs). The statistical significance level was set at 0.05, and all tests were two-sided.

## Results

### PRRT cohort characteristics

The general demographic characteristics of the full PRRT cohort are included in Table 1. We identified a total of 173 patients receiving PRRT with ^177^Lu-DOTATATE on a clinical trial or as standard of care. The earliest treatment date was 03/2017, and the latest was 12/2022. The total cohort had a nearly balanced distribution by sex (48.6% male) and a median age of 65 years at PRRT initiation. The majority of patients received four PRRT fractions (77.5%), and only one patient received five fractions.

**Table 1:** Patient characteristics (whole cohort).

Characteristic	Overall
	(*n* = 173)
Sex	
Female	89 (51.4%)
Male	84 (48.6%)
Age	173, 65, (24, 93), 64.1 (11.5)
Primary site	
GI	145 (83.8%)
GU	2 (1.2%)
Bronchopulmonary	14 (8.1%)
Female reproductive and breast	1 (0.6%)
Others/unknown	11 (6.4%)
Ki-67	145, 7.0, (0.4, 60.0), 9.3 (8.8)
Missing	28 (16.2%)
Grade	
1	37 (21.4%)
2	104 (60.1%)
3	11 (6.4%)
Unknown	20 (11.6%)
Missing	1 (0.6%)
PRRT fractions	
1	16 (9.2%)
2	14 (8.1%)
3	8 (4.6%)
4	134 (77.5%)
5	1 (0.6%)

*n*, median, (min, max), mean (SD) for continuous variables; *n* (%) for categorical variables.

### Myelodysplasia cohort characteristics

A total of 10/173 (5.8%) patients (4 males and 6 females) were diagnosed with tMNs at a median of 23 months (5.63) after the first PRRT cycle. Another one developed severe thrombocytopenia, stopped all treatments and was lost to follow-up; that patient was not included in our analysis. Table 2 summarizes the basic demographic characteristics of our myelodysplasia cohort. Four out of the 10 patients were male (40%), and 6 (60%) were female. Most patients (90%) had gastroenteropancreatic neuroendocrine neoplasms, and only one was reported to have well-differentiated high-grade (Ki-67 up to 21%) disease. The median age at PRRT initiation was 68 years (range: 24–78), and most (77.5%) patients received all four fractions to a total of 800 mCi. All patients were previously treated with somatostatin analogs (SSAs), but most (90%) had at least two prior treatments before PRRT was used. Two (20%) of the patients had received chemotherapy before PRRT, and one had received capecitabine and temozolomide (developed MDS). Treatments following PRRT were mostly SSAs (40%) or observation/no treatment (40%) with only one patient being placed on a clinical trial and one patient receiving chemotherapy with capecitabine and temozolomide (developed AML).

**Table 2:** Demographic and patient baseline characteristics (patients with MDS/AML).

Variable	No. of patients
Gender	
Male	4 (40%)
Female	6 (60%)
Age at PRRT initiation	
>50 years old	8 (80%)
<50 years old	2 (20%)
Primary tumor site	
Stomach	1 (10%)
Pancreas	2 (20%)
Bowel	6 (60%)
Pelvic sidewall	1 (10%)
Grade/Ki-67 range	
Low grade, <3%	5 (50%)
Intermediate grade, 3–20%	4 (40%)
High grade (>20%)	1 (10%)

Total tumor volume (TTV) and total lesion somatostatin receptor expression (TL-SSTR) were quantified using PET-based volumetric analysis in patients who developed AML/MDS (Table 3). Baseline TTV ranged from 7.9 to 286.3 mL. Four patients had low tumor volume (<54.9 mL), and six patients had high tumor volume (≥54.9 mL). Overall, myelodysplasia occurred across the full spectrum of baseline tumor volume in these 10 patients.

### AML/MDS characteristics

The follow-up was extended to 87 months from PRRT initiation with data collected until January 2025. The median follow-up was 36 months. Two patients developed AML, six developed MDS, one developed aplastic anemia, and one developed multilineage dyspoiesis, but these latter cases were reclassified as MDS. Cases of AML were diagnosed after 2 and 4 years from the beginning of treatment, multilineage dyspoiesis at 5 years, while the overwhelming majority of MDS cases were diagnosed by 1 year after initiation of treatment. Cytogenetics are included in Table 4.

**Table 3:** Tumor volume characteristics.

Identification number	Tumor volume	Total tumor volume (mL)	TL-SSTR
1	High	286.3	4,830.2
2	High	128.6	1,705.1
3	Low	50.1	726
4	Low	25.1	361.3
5	High	150.2	2,757.7
6	Low	7.9	116.4
7	High	180.1	3,647.1
8	High	95.9	2,083.9
9	Low	18.1	139.6
10	High	144.2	2,184.5

TTV, total tumor volume; TL-SSTR, total lesion somatostatin receptor expression. Four patients had low tumor volume, and six patients had high tumor volume.

**Table 4:** Tumor and treatment characteristics, latency periods and survival rates.

ID	Treatments prior to PRRT	Cytogenetics on NEN	Latency period (beginning of treatment to MDS dx)	*n* of Fx	Total delivered	Treatments after PRRT	AML/MDS type 2	Cytogenetics on MDS/AML	WHO 5th edition classification	ICC 2022	ELN 2022	IPSS-R	Treatment	Time survived with hematological abnormality by 01/2025	Alive or dead
1	Octreotide, radioembolization, chemoembolization	VUS: MET c.1085T>C	4 years and 10 months	3	600	SBRT, octreotide	M2	Monosomy/deletion of ETV6 (12p) – 32.5%/RUNX1T1/RUNX1 rearrangement – 46%/molecular panel negative/post-induction new DNMT3A VUS but RUNX negative	Acute myeloid leukemia with RUNX1:RUNX1T1 fusion	AML with t(8; 21)(q22; q22.1)/RUNX1:RUNX1T1 ≥ 10%	Favorable	NA	7 + 3, gemtuzumab ozogamicin and HiDAC consolidation	35 months	Alive
2	Octreotide, everolimus	N/A	5 years and 3 months	4	800	RT to liver, cabozantinib on clinical trial	Low grade MDS	Trisomy 8/RUNX1T1 (FISH negative for RUNX1T1/RUNX1 rearrangement)	MDS with low blasts	MDS, NOS with multilineage dysplasia	NA	Low	Supportive	6 months	Dead
3	Lanreotide, everolimus	N/A	2 years and 3 months	4	800	Capecitabine and temozolomide	T-AML	Monosomy 7, subsequent VUS in PTPN11, BRAF and PHF6 mutation	AML, myelodysplasia related	AML with myelodysplasia related gene mutations	Unfavorable	NA	DD venetoclax, Vyxeos, hydroxyurea, fludarabine/Cy CIML NK trial (haploidentical transplant from family member), FLAG-IDA	8 months	Dead
4	Octreotide	Normal	9 months	4	800	None	Aplastic anemia	Normal	MDS with low blasts	MDS, NOS with multilineage dysplasia	NA	Intermediate	Supportive	16 months	Dead
5	Gemcitabine and oxaliplatin, 5-FU, radioembolization, clinical trial with bevacizumab, lanreotide, TACE, everolimus	N/A	9 months	2	800	None	MDS-RS	FISH negative, Myeloseq: IDH2 R140Q, SRSF2 P95H, ASXL1 E865Dfs*2, RUNX1 A324Lfs*7 and L98Sfs*24/VUS TET2, NF1	MDS with low blasts	MDS, NOS with single lineage dysplasia	NA	Low	None	8 months	Dead
6	RFA to liver, octreotide, everolimus, capecitabine and temozolomide	N/A	5 months	4	800	None	MDS	Myeloseq: SF3B1 K700E, ASXL1 G646Wfs*12, TP53 M235I, VUS: DNMT3A (3 instances), SUZ12, PPM1D	MDS with low blasts and SF3B1 mutation	MDS with mutated SF3B1	NA	Intermediate	None	4 months	Dead
7	Lanreotide, everolimus	N/A	1 year and 7 months	4	3,200	None	High risk MDS	FISH: MECOM rearrangement with extra 3′MECOM, deletion of 7q and monosomy 7	AML with MECOM rearrangement	AML with inv(3)(q21.3q26.2) or t(3; 3)(q21.3; q26.2)/GATA2; MECOM(EVI1) ≥ 10%	Unfavorable	NA	None	6 months	Dead
8	Temsirolimus and bevacizumab, octreotide, TACE, radioembolization, X82 with everolimus	DAXX c1501+1G>C, MEN1 W126fs, MLL2 Q3764*/VUS: DNMR3A, SETBP1, AKT3, MEN1	11 months	4	800	SSAs (lanreotide)	Intermediate grade MDS	Monosomy 7, no abnormalities on Myeloseq	MDS with increased blasts 2	MDS with excess blasts	NA	Very high	None	27 months	Dead
9	Octreotide, everolimus	N/A	4 years 4 months	4	800	SSAs	AML	FISH: Monosomy 5 (D5S630/D5S2064/EGR1), deletion of CBFB, monosomy 17 (TP53/D17Z1), deletion of TP53 (17p), monosomy RARA (17q)	AML, myelodysplasia related	AML with mutated TP53	Unfavorable	NA	Decitabine + venetoclax	15 months	Dead
10	Octreotide, bland embolization	No variants of significance but BRCA2 germline mutation	3 years 1 month	4	800	Chemoembolization, SSAs	MDS	Chromosome: Clonal aberrations in chromosome 13 FISH: Deletion of D13S319	MDS with low blasts	MDS, NOS with single lineage dysplasia	NA	Intermediate	None	17 months	Alive

AML, acute myeloid leukemia; ELN, European Leukemia Network; Fx, fractions; ICC, International Consensus Classification; MDS, myelodysplastic syndrome; IPSS-R, International Prognosis Scoring System – Revised; MDS-RS, myelodysplastic syndrome with ringed sideroblasts; NEN, neuroendocrine neoplasia; PRRT, peptide receptor radionuclide therapy; RFA, radiofrequency ablation; RT, radiotherapy; SBRT, stereotactic body radiotherapy; SSA, somatostatin analog; TACE, transarterial chemoembolization; VUS, variants of uncertain significance; 5-FU, 5-fluorouracil.

Overall, due to limited sample size, determination of common pathways was not feasible. However, 50% (5/10) of the patients had monosomies, with 30% (3/10) presenting with chromosome 7 monosomy. Of the patients with hematological abnormalities, three received cytotoxic chemotherapy for leukemia, while 70% received supportive care or no treatment. Eight patients died (80%) after a median duration of 8 months (4.23) from tMN diagnosis. Of the three patients who received treatment, one (33.3%) was alive as of last review. Of the seven patients who were placed on supportive care or observation, one (14.3%) was alive as of last review. As seen in Table 4, cytogenetic and gene findings were heterogeneous and no specific cytogenomic findings were overrepresented.

**Figure 1:**
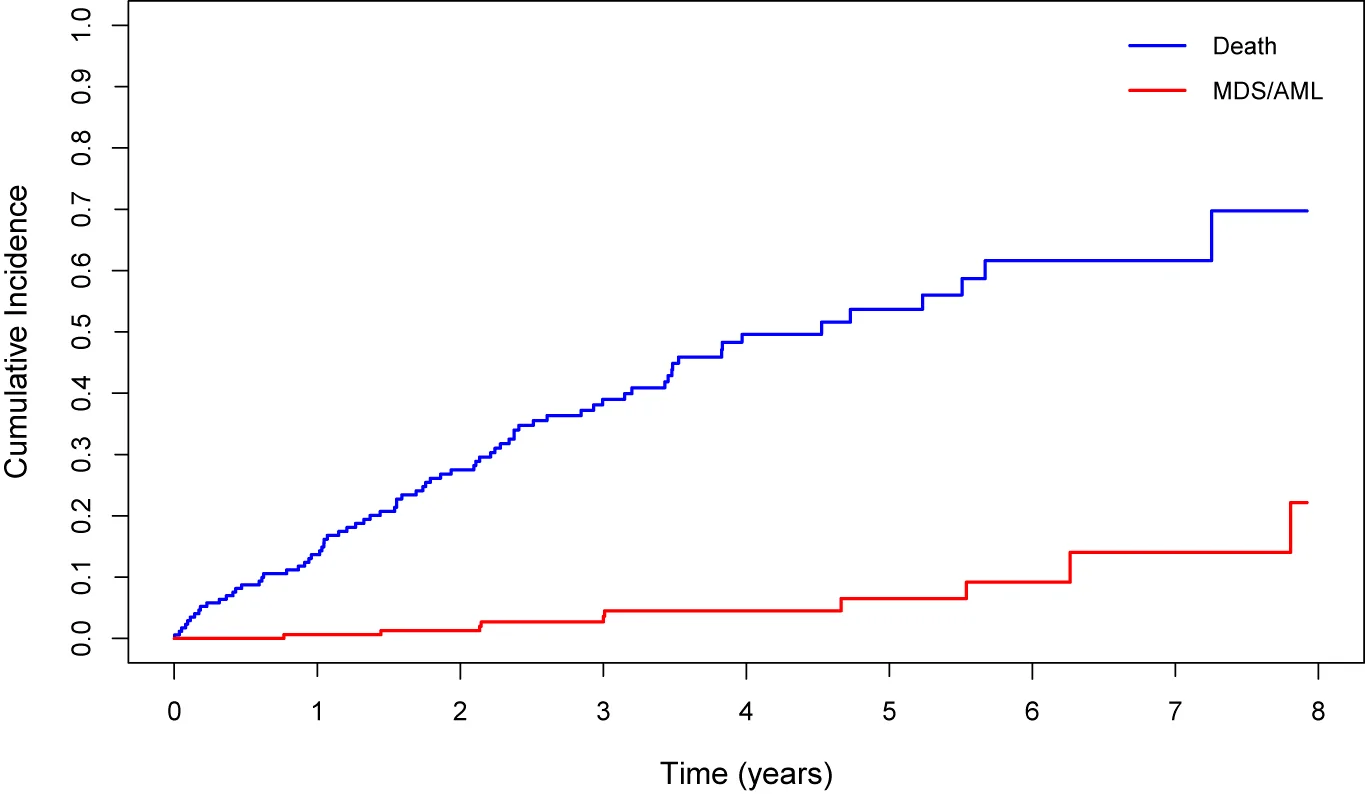
Cumulative incidence of MDS/AML and death.

The probability of MDS/AML increased with time from the end of PRRT from 0.006 (95% CI: 0.001–0.031) on year 1 to 0.141 (95% CI: 0.049–0.278) on year 7 (Table 5 and Fig. 1). The median time to death was 4.53 years (95% CI: 3.45 to not reached). A multivariable model showed that female gender was associated with a higher risk of death (1.685, 95% CI: 1.058–2.686, *P* = 0.028), while there was no sex preponderance for MDS/AML (data not shown).

**Table 5:** Cumulative incidence of death and MDS/AML.

Year	Death		MDS/AML	
	Probability	95% CI	Probability	95% CI
1	0.137	(0.090, 0.193)	0.006	(0.001, 0.031)
2	0.275	(0.208, 0.346)	0.013	(0.003, 0.042)
3	0.390	(0.310, 0.469)	0.027	(0.009, 0.063)
4	0.496	(0.403, 0.582)	0.045	(0.018, 0.091)
5	0.537	(0.433, 0.630)	0.065	(0.025, 0.131)
6	0.616	(0.487, 0.722)	0.092	(0.036, 0.181)
7	0.616	(0.487, 0.722)	0.141	(0.049, 0.278)

## Discussion

PRRT achieves a high response rate and prolongs PFS in patients with somatostatin receptor-positive gastrointestinal NENs^1(p1),3^. Although the real-world long-term incidence and outcomes of hematological abnormalities have been reported in both prospective and retrospective studies, there is no true consensus on the frequency of tMNs in real world. Our study identified 10 eligible out of 173 treated patients, raising the percentage of hematologic abnormalities to 5.8%, notably higher than the rate of 2% reported in a phase 3 study (7). Moreover, Fig. 1 shows that cumulative incidence rates increased from 1- to 5-year follow-up, more than doubling in a 60-month follow-up period. This could be related to the fact that some of our initial patients had multiple treatments before PRRT was approved as a standard of care with effect on their bone marrow reserves. We plan to review patient outcomes again after 2 more years have passed and determine whether the rates of MDS/AML remain the same after the initial backlog of patients has been treated and more eligible individuals receive PRRT as a second- or third-line treatment. We also feel that our numbers could reflect a closer follow-up in our institution with more strict criteria for diagnosis of a hematological disorder (two patients were reclassified as having a tMN). Finally, there might be an underdiagnosis problem in patients with more subtle complete blood count abnormalities outside the academic setting. MDS, especially low-grade one, might remain undetected in the community or attributed to later-line treatments or organ (e.g. liver and kidney) dysfunction.

The latency period for the occurrence of tMNs in our study is consistent with the published literature. Studies have shown a median time for persistent hematologic dysfunction development of 41 months after the first PRRT cycle (9). A large cohort study of 1,631 patients who received PRRT between 1999 and 2019 reported a median latency period of 43 months (ranging from 6 to 123 months) from the first PRRT to the diagnosis of tMNs (6). The variation in this last study suggests the presence of individual predisposition factors that may influence the timeline of disease development. Consistent with previous papers, we encountered cases suggesting that chemotherapy or radiotherapy exposure, either before or after PRRT, may contribute to the development of AML/MDS. Within our cohort, three patients had exposure to antimetabolites and platinum agents (Table 4) – one was treated with gemcitabine and oxaliplatin and the other two were treated with capecitabine and temozolomide. The two patients that received chemotherapy before PRRT had shorter latency periods (5 and 9 months, respectively). A prospective study of PRRT with capecitabine and temozolomide (11) reported a higher rate of myelodysplasia and longer latency periods, although there was no appreciable difference between PRRT only and chemoradiation patients. Four patients were previously treated with either chemoembolization or radioembolization. The combination of chemotherapy and radiation therapy has been associated with a heightened risk of developing secondary leukemia, especially with alkylating agents alone (9), combined radiation and drug therapy (12) or sequential treatments (13).

We conducted a non-systematic PubMed search (Table 6), performed in January 2025, identifying 15 relevant articles (6, 8, 9, 14, 15, 16, 17, 18, 19, 20, 21, 22, 23, 24, 25). The MDS/AML incidence rate in our study is higher than in most of the reviewed studies, with the exception of Kennedy *et al.* (19), who reported an incidence rate of 6.7% with an extended follow-up period of 68 months. Two studies (22, 23) reported no MDS/AML incidence; however, noted small sample sizes might have affected that. The median overall survival after myelodysplasia diagnosis was only documented in four studies and ranged from 4 to 14.4 months. In our study, some patients remain on follow-up, so the median OS has not been reported. All the reviewed studies provided data on prior chemotherapy or radiotherapy exposure in their cohorts. However, information on genetic mutations was scarce, with only three studies reporting such data.

**Table 6:** Myelodysplasia rates, survival characteristics and follow-up periods in similar studies.

Author	Sample size (patients)	Myelodysplasia/leukemia rates (%)	Median overall survival (months)	Median follow-up period (months)	Median overall survival after myelodysplasia (months)	Data on prior chemotherapy or radiotherapy	Data on genetic mutations
Garske-Román *et al.*	200	2	43	31	-	Yes	No
Baum *et al.*	1,048	2.10	51	-	14.4	Yes	No
Fröss-Baron *et al.*	102	1	42	34	-	Yes	No
Bergsma *et al.*	367	4	-	29	-	Yes	No
Goncalves *et al.*	521	4.80	62	51	13	Yes	Yes
Singh *et al.*	13	-	-	-	-	-	Yes
Kennedy *et al.*	104	6.70	71	68	-	Yes	No
van der Zwan *et al.*	181	2.20	80.8	88.6	-	Yes	No
Bodei *et al.*	807	2.35	-	30	-	Yes	No
Chantadisai *et al.*	1,631	1.83	-	55	13	Yes	Yes
Kong *et al.*	27	0	81	67	-	Yes	No
Jiang *et al.*	30	0	-	46	-	Yes	No
Brieau *et al.*	20	20*	-	37	4	Yes	No
Sabet *et al.*	203	1.40	-	31	-	Yes	No
Brabander *et al.*	610	2.20	63	78	-	Yes	No

* Cohort of patients heavily pretreated with alkylating chemotherapy.

We were unable to note a particular trend with regard to tumor volume, as it was evenly distributed among our patients. However, no such associations have been reported in the literature. A multi-center study in Germany did not identify a significant relationship between tumor burden and the occurrence of general adverse effects, indicating that a higher tumor load may not necessarily predict increased treatment-related toxicity (15). Furthermore, a recent study assessing the risk of hepatotoxicity in patients undergoing PRRT treatment that specifically examined patients with a very high tumor burden indicated that even in patients with extensive organ involvement, PRRT did not result in significant toxicity (26).

Our study reports findings of both institutional immunohistochemistry and cytology in our diagnosed patients, and a notable finding was the presence of monosomies, including monosomy 7 in 3 out of 10 patients who developed myelodysplasia or leukemia (Table 4). Monosomy 7 has been strongly associated with therapy-related leukemia, particularly following radiation exposure and alkylating chemotherapy agents. Previous literature supports a link between radiation therapy and the development of monosomy 7 abnormalities (27), reinforcing the hypothesis that radiation-related DNA damage may have contributed to leukemogenesis in these cases. A study by Singh *et al.* (18) analyzed patients with neuroendocrine tumors undergoing PRRT and found that 62% had clonal hematopoiesis (CH) at baseline. Notably, after PRRT exposure, there was a clonal expansion of mutations in DNA damage response genes, including TP53, accompanied by cytopenias in 75% of the patients studied. This suggests that PRRT may select for hematopoietic clones harboring TP53 mutations, contributing to hematologic toxicity and potential progression to tMNs. Other studies reported that, among patients who developed tMNs following PRRT, 36% had TP53 mutations (17) , similarly to the 20% found in our cohort. Moreover, three patients were found to have RUNX1 mutations. While direct evidence linking RUNX1 mutations specifically to PRRT-induced myelodysplasia is limited, RUNX1 mutations are well established in therapy-related myeloid neoplasms (tMNs), including myelodysplastic syndrome (MDS) and acute myeloid leukemia (AML) (27, 28, 29). Given the role of RUNX1 mutations in other therapy-related myeloid neoplasms, it is plausible that similar mechanisms could contribute to PRRT-induced myelodysplasia. Further research is needed to establish a direct causal relationship and to understand the underlying mechanisms.

At the time of this analysis, eight patients had succumbed to disease, while two remained alive (Table 4). In patients who received antileukemic treatments, survivals ranged from 8 to 35 months, which is inconsistent with the generally dismal outcomes seen in the literature. It is not known if treatment at a tertiary care center can contribute to improved survival or if our numbers are skewed by patients with lower aggressiveness MDS – the numbers are too small to determine.

In conclusion, this study suggests a higher than reported incidence of therapy-related myeloid neoplasms following PRRT, with a potential link to radiation-induced genetic abnormalities. This calls for careful sequencing of radioligand therapies in patients with SSTR-positive NENs.

## Declaration of interest

The authors have no relevant financial or non-financial interest to disclose.

## Funding

This work did not receive any specific grant from any funding agency in the public, commercial or not-for-profit sector.

## Author contribution statement

NAT conceived the study. JPTB, NAT, SW and JL created the first version of the manuscript. SW and JL numerically analyzed the data. All authors reviewed the data and edited the subsequent and final versions of the manuscript.
